# Effects of feeding different proportions of steam-flaked corn-based starter on growth performance, immunity and serum metabolism of pre-weaned Simmental calves

**DOI:** 10.3389/fvets.2024.1502738

**Published:** 2025-01-14

**Authors:** Kangyu Yao, Yu Gao, Liang Yang, Min Yang, Xiuyu Wu, Xinyu Zhang, Zhengke Lv, Wei Shao, Yong Wei, Wanping Ren

**Affiliations:** College of Animal Science, Xinjiang Agricultural University, Urumqi, China

**Keywords:** calf, growth performance, immunity, serum biochemical indicator, metabolomics

## Abstract

**Introduction:**

This study examines the effects of steam-flaked corn starter on pre-weaned Simmental calves' growth, immunity, and metabolism. Despite benefits shown in adult cattle, research on calves is limited. The goal is to optimize calf feeding for better growth, health, and nutrient use.

**Methods:**

Thirty-two Simmental bull calves (avg. wt: 50.50 ± 4.50 kg, avg. age: 21 ± 7 days) were divided into four groups of eight. The 127-day study included a 7-day pretest and a 120-day trial. Calves had unlimited access to starter feed, alfalfa hay, and water. Groups received starter diets with 0% (CK, control), 33% (SFC33, low), 66% (SFC66, medium), or 100% (SFC100, high) steam-flaked corn replacing regular corn. Other conditions were consistent.

**Results:**

Feeding 33% steam-flaked corn to pre-weaned Simmental calves led to highest daily weight gain (0.80 kg/d), significantly boosting serum globulin, cholesterol, urea nitrogen, glucose, immunoglobulins, GH, INS, and IGF-1 (*P* < 0.05). Compared to control, 31 metabolites differed in SFC33 group, mostly up-regulated, including glycerophospholipids, linoleic/arachidonic acid metabolism, cholesterol pathway molecules, L-glutamine in multiple pathways, and mannose in fructose/mannose metabolism.

**Discussion:**

In summary, feeding 33% steam-flaked corn-based starter can improve the growth performance, enhance immunity, and improve sugars, lipids, and proteins metabolism of pre-weaned Simmental calves.

## 1 Introduction

The health of calves is vital to the entire livestock industry, and their wellbeing directly impacts the efficiency and long-term development of the farm. Among the numerous influencing factors, nutritional elements play a central role. Proper nutrition is crucial for healthy calf growth, impacting their physical development, immunity, and future production potential and performance (1, 2). The digestive and immune systems of pre-weaned calves are in a phase of rapid development, and nurturing at this stage plays a crucial role in the systematic establishment of calf digestion and immunity, as well as being decisive for subsequent production potential (3).

Steam-flaked corn is a high-quality energy feed. The process of steam-flaking corn enhances the content of rumen-degradable starch, which is more easily digested in the rumen. Compared to ordinary crushed corn, the rapid degradation of steam-flaked corn in the rumen increases the concentration of rumen volatile fatty acids (VFA) (4, 5). This allows cattle to access the same nutritional value while consuming less feed. Using steam-flaked corn in dairy cattle feeding can increase milk production and reduce somatic cell count, effectively improving the quality of milk (6, 7); in beef cattle feeding, using steam-flaked corn as feed can significantly improve the productivity and health of the cattle. This can lead to better serum biochemical indexes, promoting healthy growth and efficient output (8, 9). However, there are relatively few studies on calf feeding, so in this experiment, pre-weaned Simmental calves were selected as the test subjects and steam-flaked corn was mixed into their starter feed in different proportions for feeding. Through analysis of the daily weight gain of the calves, as well as examination of serum biochemical indexes, hormone levels, metabolites, and metabolic pathways, our goal is to investigate the specific effects of steam-flaked corn starter on the daily weight gain, immune function, and serum metabolism of pre-weaned Simmental calves. Our objective was to systematically investigate the specific and comprehensive effects of steam-flaked corn starter on daily weight gain, immune function, and serum metabolic indexes of pre-weaned Simmental calves. We aimed to reveal the unique role of steam-roasted corn starter in calf feeding through scientific methods, and to provide a solid and reliable theoretical basis and practical guidance for further optimizing calf feeding management strategies, improving calf growth performance, enhancing their health status, and improving their nutrient metabolism.

## 2 Materials and methods

### 2.1 Study conditions

The trial was conducted from July to November 2023 at Xinjiang Nongwang Ranch Cattle Farm, Tashiu Township, Hot Spring County, Bortala Mongol Autonomous Prefecture, Xinjiang.

### 2.2 Materials

The steam-pressed corn used for making starter feed of calves was purchased from Xinjiang Bosheng Co., LTD. (Bo Le, China), the milk substitute powder used for daily brewing and feeding was purchased from Beijing Dazheng Shuangsheng Co., LTD. (Beijing, China), and the weighbridge used for weighing calves was purchased from Shanghai Yaohua Weighbridge Co., LTD. (Shanghai, China). The tape measure used to measure the body size of calves and the blood collection needle, 5 mL blood collection vessel, 2 mL freezing tube and sterile gloves used in the process of calf blood collection were purchased from Xinjiang Ziqi Biological Co., LTD. (Urumqi, China).

### 2.3 Experimental design

Thirty-two healthy Simmental bull calves with similar weights (average weight of 50.50 ± 4.50 kg) and ages (average age of 21 ± 7 days) were chosen for the study. They were divided into four groups, each with eight calves. The study consisted of a pretest phase lasting 7 days and a formal trial phase lasting 120 days. Throughout the trial, all the calves were given unrestricted access to a specific starter feed, alfalfa hay, and water. The control group (CK group) received a starter diet without steam-flaked corn, while in trial groups 1 (SFC33 group), 2 (SFC66 group), and 3 (SFC100 group). In order to explore a more efficient replacement ratio for steam- flaked corn, four levels of steam- flaked corn, 0% (control), 33% (low level), 66% (medium level), and 100% (high level), were used to replace regular crushed corn in the appetizer diets, respectively. All other experimental conditions were kept constant across the four groups.

### 2.4 Feed and management

All calves were housed individually in calf islands with starter and alfalfa hay placed in two stainless steel tubs to ensure that they had free access to the special starter and alfalfa hay throughout the trial, as detailed in Table 1. Calves had free access to water. In addition to the basal diet, milk replacer was mixed with warm water at a ratio of 1:7 in calf milk bottles and fed to each calf at 8:00 a.m. and 7:00 p.m.

**Table 1:** Formulation and nutrient composition of starter diets.

**Composition (%)/item^a^**	**CK**	**SFC33**	**SFC66**	**SFC100**
Ordinary crushed corn	42	28	14	0
Steam flaked corn	0	14	28	42
Wheat bran	15	15	15	15
Soybean meal	24	24	24	24
Whey powder	7	7	7	7
Premix^b^	1.5	1.5	1.5	1.5
Salt	0.5	0.5	0.5	0.5
Alfalfa grass powder	10	10	10	10
**Nutrient composition (DM)/%**				
CP	18.93	18.52	18.68	18.40
CF	8.23	8.67	8.69	8.45
CA	6.34	6.06	5.96	5.96
NDF	28.61	30.07	28.32	32.17
ADF	9.56	9.43	8.64	11.20
Ca	0.62	0.64	0.63	0.63
P	0.37	0.38	0.38	0.37
GE(MJ/kg)	17.62	17.82	17.76	17.62

^a^CK, control check, 0% steam-flaked corn was used to replace the original crushed corn in the starter. SFC33: 33% steam-flaked corn was used to replace the original crushed corn in the starter. SFC66: 66% steam-flaked corn was used to replace the original crushed corn in the starter diet. SFC100: 100% steam-flaked corn was used to replace the original crushed corn in the starter die, *n* = 8.

^b^Premix ingredients for vitamin A ≥ 80,000 IU, vitamin D_3_ ≥ 20,000 IU, vitamin E ≥ 500 IU, iron (ferrous sulfate) ≥ 1,100 mg, copper (basic copper chloride) 300–600 mg, zinc (zinc sulfate) 1,000-2,500 mg, manganese (manganese sulfate) ≥ 200 mg, selenium (selenium sulfate) 10 mg, iodine (iodate of calcium) ≥ 10 mg, calcium 12–18%, total phosphorus ≥3%, sodium chloride 16–20%. DM, dry matter; CP, crude protein; CF, crude fiber; CA, crude ash; NDF, neutral detergent fiber; ADF, acid detergent fiber; Ca, calcium; P, phosphorus; GE, gross energy.

### 2.5 Sample collection and measurement

Growth performance data and blood samples were collected on days 0, 40, 80, and 120 of the trial. In brief, weights of calves were measured by soft tape to analyze daily weight gain data. Blood samples were collected from the jugular vein of calves at 7:00 a.m. in a fasted state using a blood collection needle and tube, and then placed in a centrifuge at 3,500 r/min for 15 min after 1 h of resting, and then the centrifuged serum was divided into two freezing tubes, one tube was stored in a refrigerator (−20°C), and the other tube was stored in liquid nitrogen (−196°C). Finally, the collected blood samples were sent to the Huaying Institute of Biotechnology (Beijing, China) for the determination of biochemical indices in the blood serum. The serum samples from the CK and SFC33 groups on day 120 were sent to Wuhan Metabolism Company for non-targeted metabolomics studies. Feed samples were collected by five-point sampling method and then stored in a refrigerator at −20°C, and finally sent to Xinjiang Key Laboratory of Dairy Meat and Herbivore Nutrition for nutrient content testing.

### 2.6 Measurement of serum indicators

#### 2.6.1 Serum biochemical indices

TP, ALB, GLB, TG, TC, GLU, BUN were measured using the corresponding bisulfonylurea assay fully automated type (liquid) kits on a Myriad BS-420 fully automated biochemistry analyser (wavelength 546 nm, reaction temperatures of 25°C, 37°C, optical diameter of the cuvette 1 cm). Firstly, 1.0 mL of physiological saline was added to the blank tube, the standard tube and the sample tube and 12.5 μl of physiological saline, standard and sample was added in the corresponding tubes were filled with 12.5 μl of saline, standard and sample, mixed well and then held at the reaction temperature for 10 min, zeroed with a reagent blank tube, and A standard and A sample were determined, respectively, and the concentration was calculated by the formula: concentration = (A sample/A standard)^*^standard concentration (g/L).

#### 2.6.2 Immune factors

IgA, IgM, IgG were measured using (HY-755) KIT final kit in Myriad BS-420 automatic biochemistry analyser (wavelength: 610 nm for IgG, 240 nm for IgA/M, reaction temperature 37°C), the reactant R1 and the corresponding antiserum R2 were mixed into the working solution in a ratio of 3:1, and then 100 μl of standards and samples were added into the blank, standard and sample tubes, respectively. One milliliter of working solution was added into the blank tube, standard tube and sample tube, respectively, and then 100 μl of standard and sample were added into the standard tube and sample tube, respectively, after mixing and incubating at 37°C for 10 min, the wavelength of IgG: 700 nm, IgA/M: 340 nm at which the working solution was withered, and the absorbance of each tube was measured.

#### 2.6.3 Hormone level

GH, INS, IGF-1, IGF-2 were measured using ELISA KIT kits on a Huawei Drum DR-200BS enzyme labeling analyser:

(1) After the dilution of the standard in the microtiter plate add antibody 100 μl per well after 2 h of encapsulation and discard the liquid and shake dry, add 300 μl of diluted washing solution per well, oscillate for 30 s, shake off the washing solution, pat dry with absorbent paper, and so on for 5 times, pat dry.

(2) Add 200 μl of sealing solution to each well, shake gently and mix well, let it stand for 30 min at room temperature, discard the liquid, shake it off, add 300 μl of diluted washing solution to each well, oscillate for 30 s, shake off the washing solution and pat dry with absorbent paper. Repeat this 5 times and pat dry.

(3) Add 25 μl of coating solution, 25 μl of specimen, 50 μl of standard to each well, shake gently to mix, add 100 μl of HRP to each well, shake gently to mix, and let it stand at room temperature for 60 min. Discard the liquid, shake it off, and then add 300 μl of diluted washing solution to each well, shake for 30 s, shake off the washing solution, and then pat it dry with blotting paper. Repeat this 5 times and pat dry.

(4) Add 100 μl of color development solution to each well, shake gently and mix well, develop the color for 15 min, and add 100 μL of termination solution to each well to terminate the reaction (at this time, the blue color turned yellow). Measurement: Adjust the zero with the blank well, and measure the absorbance value (OD value) of each well at 450 nm. Measurement should be carried out within 15 min after the addition of termination solution.

### 2.7 Metabolomics

#### 2.7.1 Liquid samples preparation

The sample stored at −80°C refrigerator was thawed on ice and vortexed for 10 s. Fifty microliters of sample and 300 μL of extraction solution (ACN: Methanol = 1:4, V/V) containing internal standards were added into a 2 mL microcentrifuge tube. The sample was vortexed for 3 min and then centrifuged at 12,000 rpm for 10 min (4°C). Two hundred microliter of the supernatant was collected and placed in −20°C for 30 min, and then centrifuged at 12,000 rpm for 3 min (4°C). A 180 μL aliquots of supernatant were transferred for LC-MS analysis.

#### 2.7.2 HPLC conditions

All samples were for two LC/MS methods. One aliquot was analyzed using positive ion conditions and was eluted from the T3 column (Waters ACQUITY Premier HSS T3 Column 1.8 μm, 2.1 mm × 100 mm) using 0.1% formic acid in water as solvent A and 0.1% formic acid in acetonitrile as solvent B in the following gradient: 5–20% in 2 min, increased to 60% in the following 3 min, increased to 99% in 1 min, and held for 1.5 min, then come back to 5% mobile phase B within 0.1 min, held for 2.4 min. The analytical conditions were as follows, column temperature, 40°C; flow rate, 0.4 mL/min; injection volume, 4 μL; Another aliquot was using negative ion conditions and was the same as the elution gradient of positive mode.

#### 2.7.3 MS conditions (AB)

The data acquisition was operated using the information-dependent acquisition (IDA) mode using Analyst TF 1.7.1 Software (Sciex, Concord, ON, Canada). The source parameters were set as follows: ion source gas 1 (GAS1),50 psi; ion source gas 2 (GAS2), 50 psi; curtain gas (CUR), 25 psi; temperature(TEM), 550°C; declustering potential (DP), 60 V, or −60 V in positive or negative modes, respectively; and ion spray voltage floating 45 (ISVF), 5,000 V, or −4,000 V in positive or negative modes, respectively. The TOF MS scan parameters were set as follows: mass range, 50–1,000 Da; accumulation time, 200 ms; and dynamic background subtract, on. The product ion scan parameters were set as follows: mass range, 25–1,000 Da; accumulation time, 40 ms; collision energy, 30 or−30 V in positive or negative modes, respectively; collision energy spread, 15; resolution, UNIT; charge state, 1 to 1; intensity, 100 cps; exclude isotopes within 4 Da; mass tolerance, 50 ppm; maximum number of candidate ions to monitor per cycle, 18.

### 2.8 Analysis of serum metabolic data

The original data file acquisited by LC-MS was converted into mzXML format by ProteoWizard software. Peak extraction, peak alignment and retention time correction were, respectively performed by XCMS program. The “SVR” method was used to correct the peak area. The peaks with detetion rate lower than 50% in each group of samples were discarded. After that, metabolic identification information was obtained by searching the laboratory's self-built database, integrated public database, AI database and metDNA.

#### 2.8.1 PCA

Unsupervised PCA (principal component analysis) was performed by statistics function prcomp within R (http://www.r-project.org). The data was unit variance scaled before unsupervised PCA.

#### 2.8.2 Hierarchical cluster analysis and Pearson correlation coefficients

The HCA (hierarchical cluster analysis) results of samples and metabolites were presented as heatmaps with dendrograms.

#### 2.8.3 Differential metabolites selected

For two-group analysis, differential metabolites were determined by VIP (VIP > 1) and *P*-value (*P*-value < 0.05, Student's *t*-test). VIP values were extracted from OPLS-DA results, which also contain score plots and permutation plots, and were generated using the R package MetaboAnalystR. The data was log transform (log2) and mean centering before OPLS-DA. To avoid overfitting, a permutation test (200 permutations) was performed.

#### 2.8.4 KEGG annotation and enrichment analysis

Identified metabolites were annotated using the KEGG Compound database (http://www.kegg.jp/kegg/compound/), and annotated metabolites were then mapped to the KEGG Pathway database (http://www.kegg.jp/kegg/pathway.html). Significantly enriched pathways are identified with a hypergeometric test's *P*-value for a given list of metabolites.

### 2.9 Statistical analysis

Data were collated using Excel 2010 software (version 2016, Microsoft Corporation, USA), and one-way ANOVA was performed and data were tested for homogeneity of variance using SPSS19.0.0 (software version 20.0, IBM Corporation, USA), and the results were expressed as “Mean ± standard deviation”, with *P* < 0.05 indicates significant difference.

## 3 Results

### 3.1 Effect of feeding different proportions of steam-flaked corn-based starter on daily weight gain of pre-weaned Simmental calves

**Figure 1:**
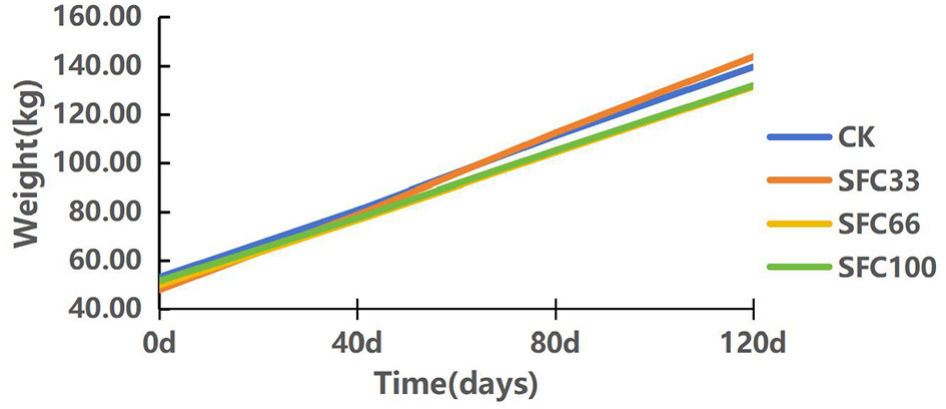
Plot of average weight change at each time point.

As shown in Table 2 and Figure 1, the SFC33 group had the highest body weight at the end of the experiment, with an average daily weight gain of 0.80 ± 0.12 kg, which was 11.11, 17.65, and 19.40% higher compared to the CK, SFC66, and SFC100 groups, respectively. The results of the experiment showed that feeding 33% steam-flaked corn-based starter increased their daily weight gain in pre-weaned calves.

**Table 2:** Effect of feeding different proportions of steam-flaked corn-based starter on daily weight gain of pre-weaned Simmental calves (kg).

**Item^a^**	**Group**				
	**CK**	**SFC33**	**SFC66**	**SFC100**	***P*** **-value**
Initial weight/kg	53.00 ± 3.56	47.75 ± 4.11	49.75 ± 4.57	51.50 ± 4.43	0.780
Final weight/kg	139.44 ± 8.83	143.85 ± 23.57	130.99 ± 11.91	132.34 ± 18.13	0.669
Net weight gain/kg	86.44 ± 9.83	96.10 ± 14.31	81.24 ± 14.87	80.84 ± 21.92	0.515
Average daily weight gain/kg·d^−1^	0.72 ± 0.08	0.80 ± 0.12	0.68 ± 0.12	0.67 ± 0.18	0.485

^a^CK, control check, 0% steam-flaked corn was used to replace the original crushed corn in the starter. SFC33: 33% steam-flaked corn was used to replace the original crushed corn in the starter. SFC66: 66% steam-flaked corn was used to replace the original crushed corn in the starter diet. SFC100: 100% steam-flaked corn was used to replace the original crushed corn in the starter diet. The results were expressed as mean ± standard deviation, *n* = 8.

### 3.2 Effect of feeding different proportions of steam-flaked corn-based starter on serum biochemical indices in pre-weaned Simmental calves

As shown in Table 3, at 40 d, the total protein level of SFC66 and SFC100 groups was significantly higher than that of the other two groups (*P* < 0.05); at 80 d, the SFC33 group was significantly higher than the other three groups (*P* < 0.05). At 80 d the albumin level of the SFC33 group was highly significantly higher than that of the SFC66 and SFC100 groups (*P* < 0.01), and higher than that of the CK group but the difference was not significant (*P* > 0.05); at 120 d, the CK and SFC33 groups were highly significantly higher than that of the SFC100 group (*P* < 0.01). At 40 d, the globulin level of the SFC100 group was significantly higher than that of the other three groups (*P* < 0.05); at 80 d, the SFC33 group was extremely significantly higher than that of the CK group (*P* < 0.01), and significantly higher than that of the SFC66 and SFC100 groups (*P* < 0.05). At 40 d, triglyceride levels were significantly higher in the SFC33 group than in the other three groups (*P* < 0.05). At 80 d the cholesterol levels in the CK and SFC33 groups were highly significantly higher than those in the SFC100 group (*P* < 0.01) and significantly higher than those in the SFC66 group (*P* < 0.05); at 120 d the CK group was highly significantly higher than the three experimental groups (*P* < 0.01). At 40 and 80 d, the urea nitrogen level of the SFC33 group was significantly higher than that of the CK group (*P* < 0.05); at 80 d, the SFC66 group was significantly lower than that of the other three groups (*P* < 0.05); at 120 d, the SFC66 group was significantly higher than that of the CK group and the SFC33 group (*P* < 0.05). At 120 d, the blood glucose levels of the test groups were all significantly higher than those of the CK group (*P* < 0.05). The results of the experiment showed that feeding 33% steam-flaked corn-based starter increased the serum levels of total protein (TP), globulin (GLB), cholesterol (TC), urea nitrogen (BUN), and glucose (GLU), and lowered the serum levels of albumin (ALB) to some extent in the pre-weaned calves. There was a significant interaction effect between group and time for TP and BUN (*P* < 0.05), and no interaction effect between group and time for the remaining indicators.

**Table 3:** Effect of feeding different proportions of steam-flaked corn-based starter on serum biochemical indices of pre-weaned Simmental calves.

**Item^a^**	**Days**	**Group**				***P*** **-value**		
		**CK**	**SFC33**	**SFC66**	**SFC100**	**Group**	**Days**	**Group** × **time**
TP (g/L)	0 d	57.06 ± 1.31	56.65 ± 2.29	53.63 ± 3.66	57.32 ± 5.32	0.148	0.000	0.002
	40 d	58.72 ± 2.13^b^	62.92 ± 7.06^b^	67.95 ± 10.93^a^	67.80 ± 10.86^a^	0.117		
	80 d	66.85 ± 4.48^b^	77.41 ± 5.71^a^	66.97 ± 5.79^b^	66.41 ± 2.28^b^	0.000		
	120 d	64.32 ± 6.01	66.12 ± 3.39	65.35 ± 8.79	63.84 ± 3.22	0.862		
ALB (g/L)	0 d	31.94 ± 1.45	30.97 ± 2.28	30.86 ± 2.24	31.55 ± 1.51	0.644	0.000	0.365
	40 d	34.04 ± 0.45	32.41 ± 3.23	30.91 ± 11.31	32.61 ± 2.72	0.360		
	80 d	37.30 ± 2.20^AB^	39.51 ± 1.43^A^	35.42 ± 0.30^B^	34.50 ± 2.72^BC^	0.000		
	120 d	37.06 ± 2.77^A^	36.48 ± 1.90^A^	35.36 ± 1.92^AB^	32.74 ± 3.33^B^	0.011		
GLB (g/L)	0 d	25.23 ± 0.54	25.66 ± 3.37	23.15 ± 3.04	25.77 ± 3.97	0.290	0.000	0.078
	40 d	24.68 ± 2.38^b^	31.22 ± 9.01^b^	32.91 ± 8.73^b^	34.14 ± 11.30^a^	0.107		
	80 d	29.56 ± 2.31^B^	37.87 ± 4.46^A^	31.56 ± 5.79^AB^	31.94 ± 4.44^AB^	0.005		
	120 d	27.31 ± 3.43	29.41 ± 2.43	29.99 ± 7.24	31.10 ± 3.58	0.417		
TG (mmol/L)	0 d	0.20 ± 0.04	0.23 ± 0.09	0.15 ± 0.02	0.17 ± 0.06	0.030	0.000	0.199
	40 d	0.22 ± 0.05^ab^	0.25 ± 0.01^a^	0.18 ± 0.09^b^	0.18 ± 0.03^b^	0.072		
	80 d	0.28 ± 0.07	0.27 ± 0.08	0.28 ± 0.03	0.24 ± 0.03	0.304		
	120 d	0.19 ± 0.08	0.21 ± 0.01	0.22 ± 0.05	0.18 ± 0.01	0.355		
TC (mmol/L)	0 d	3.12 ± 0.74	3.06 ± 0.59	3.16 ± 0.95	3.01 ± 0.93	0.981	0.001	0.222
	40 d	3.05 ± 0.65	2.89 ± 1.03	2.48 ± 0.47	2.61 ± 0.24	0.322		
	80 d	3.57 ± 0.38^A^	3.58 ± 0.19^A^	2.87 ± 0.06^B^	3.25 ± 0.38^AB^	0.000		
	120 d	3.52 ± 0.67^A^	2.74 ± 0.45^B^	2.74 ± 0.25^B^	2.42 ± 0.31^B^	0.000		
BUN (mg/dL)	0 d	5.30 ± 1.40	7.64 ± 3.49	4.89 ± 0.75	6.37 ± 1.98	0.072	0.000	0.006
	40 d	13.24 ± 1.23^b^	15.29 ± 1.07^a^	14.59 ± 2.55^ab^	14.12 ± 1.01^ab^	0.095		
	80 d	14.23 ± 0.96^a^	14.29 ± 0.87^a^	13.04 ± 0.88^b^	14.06 ± 1.70^a^	0.132		
	120 d	15.76 ± 1.03^b^	16.46 ± 1.28^b^	19.61 ± 5.18^a^	17.55 ± 2.43^ab^	0.062		
GLU (mmol/L)	0 d	3.20 ± 0.66	3.07 ± 0.54	3.11 ± 0.21	3.08 ± 0.56	0.955	0.000	0.067
	40 d	4.35 ± 0.20	3.83 ± 0.66	3.84 ± 0.78	4.08 ± 0.42	0.222		
	80 d	4.85 ± 0.31	4.61 ± 0.14	4.55 ± 0.51	4.74 ± 0.37	0.358		
	120 d	3.61 ± 2.19^b^	4.93 ± 0.26^a^	4.34 ± 0.25^a^	3.98 ± 0.98^a^	0.189		

^a^CK, control check, 0% steam-flaked corn was used to replace the original crushed corn in the starter. SFC33: 33% steam-flaked corn was used to replace the original crushed corn in the starter. SFC66: 66% steam-flaked corn was used to replace the original crushed corn in the starter diet. SFC100: 100% steam-flaked corn was used to replace the original crushed corn in the starter diet. The results were expressed as mean ± standard deviation, *n* = 8.

^A, B, C^Indicates that the difference between indicators in the same row is highly significant (*P* < 0.01).

^a, b^Indicates that the difference between indicators in the same row is significant (*P* < 0.05).

TP, total protein; ALB, albumin; GLB, globulin; TG, triglyceride; TC, total cholesterol; BUN, blood urea nitrogen; GLU, glucose.

### 3.3 Effect of feeding different proportions of steam-flaked corn-based starter on immune factors in pre-weaned Simmental calves

As shown in Table 4, at 40 d, the IgA level of SFC66 group was extremely significantly higher than that of CK group (*P* < 0.01), and that of SFC100 group was significantly higher than that of CK group (*P* < 0.05); and at 120 d, the SFC33 group was extremely significantly higher than that of SFC100 group, and significantly higher than that of SFC66 group. At 40 d, the IgM level of the SFC100 group was extremely significantly higher than that of the CK group (*P* < 0.01), and that of the SFC66 group was significantly higher than that of the CK group (*P* < 0.05); at 80 d, the SFC33 group was higher than that of the other three groups (*P* > 0.05), and significantly higher than that of the SFC100 group (*P* < 0.05); at 120 d, the SFC33 group was extremely significantly higher than that of the CK group and the SFC100 group (*P* < 0.01). At 40 d, the IgG level of SFC100 group was significantly higher than that of CK group and SFC33 group (*P* < 0.05), and SFC66 group was significantly higher than that of CK group (*P* < 0.05); at 80 d, the IgG level of SFC33 group was significantly higher than that of SFC100 group (*P* < 0.01), and significantly higher than that of the other CK and SFC66 groups (*P* < 0.05); at 120 d, SFC33 group was significantly higher than the other CK and SFC66 groups (*P* < 0.05); at 120 d, SFC33 group was significantly higher than the other CK and SFC66 groups (*P* < 0.05). At 120 d, the SFC33 group was extremely significantly higher than the other three groups (*P* < 0.01), and the CK group was significantly higher than the SFC100 group (*P* < 0.05). The results of the experiment showed that feeding 33% steam-flaked corn-based starter to pre-weaned calves significantly increased their serum levels of immunoglobulin A (IgA), immunoglobulin M (IgM), and immunoglobulin G (IgG). IgA, IgM and IgG had highly significant interaction effects between time and group (*P* < 0.01).

**Table 4:** Effect of feeding different proportions of steam-flaked corn-based starter on immune factors in pre-weaned Simmental calves.

**Item^a^**	**Days**	**Group**				***P*** **-value**		
		**CK**	**SFC33**	**SFC66**	**SFC100**	**Group**	**Days**	**Group** × **time**
IgA (g/L)	0 d	1.04 ± 0.34	0.95 ± 0.15	0.93 ± 0.10	1.15 ± 0.60	0.035	0.000	0.004
	40 d	1.03 ± 0.20^B^	1.52 ± 0.59^AB^	1.82 ± 0.59^A^	1.69 ± 0.65^AB^	0.035		
	80 d	2.19 ± 0.77	2.19 ± 0.15	2.15 ± 0.57	2.35 ± 0.37	0.870		
	120 d	2.11 ± 0.76^AB^	2.44 ± 0.19^A^	1.85 ± 0.23^AB^	1.76 ± 0.40^B^	0.026		
IgM (g/L)	0 d	7.21 ± 2.69	7.10 ± 1.69	7.16 ± 0.93	7.38 ± 4.46	0.028	0.000	0.000
	40 d	8.23 ± 2.12^B^	8.90 ± 3.27^AB^	12.16 ± 3.93^AB^	13.07 ± 3.78^A^	0.016		
	80 d	15.70 ± 4.40^ab^	18.08 ± 1.19^a^	15.60 ± 4.09^ab^	14.32 ± 1.90^b^	0.140		
	120 d	13.71 ± 3.58^B^	18.50 ± 1.20^A^	15.03 ± 3.00^AB^	13.42 ± 2.67^B^	0.003		
IgG (g/L)	0 d	0.96 ± 0.16	0.97 ± 0.21	0.93 ± 0.23	0.95 ± 0.25	0.351	0.000	0.000
	40 d	0.89 ± 0.25^B^	1.05 ± 0.19^B^	1.40 ± 0.57^AB^	1.55 ± 0.57^Aa^	0.017		
	80 d	1.63 ± 0.58^Aab^	1.79 ± 0.17^Aa^	1.41 ± 0.19^ABb^	1.14 ± 0.10^Bb^	0.003		
	120 d	1.49 ± 0.35^Bb^	2.01 ± 0.50^Aa^	1.19 ± 022^Bbc^	1.12 ± 0.48^Bc^	0.000		

^a^CK, control check, 0% steam-flaked corn was used to replace the original crushed corn in the starter. SFC33: 33% steam-flaked corn was used to replace the original crushed corn in the starter. SFC66: 66% steam-flaked corn was used to replace the original crushed corn in the starter diet. SFC100: 100% steam-flaked corn was used to replace the original crushed corn in the starter diet. the results were expressed as mean ± standard deviation, *n* = 8.

^A, B^Indicates that the difference between indicators in the same row is highly significant (*P* < 0.01).

^a, b, c^Indicates that the difference between indicators in the same row is significant (*P* < 0.05). Only comparisons between the same rows are performed.

IgA, Immunoglobulin A; IgM, Immunoglobulin M; IgG, Immunoglobulin G.

### 3.4 Effect of feeding different proportions of steam-flaked corn-based starter on hormonal changes in pre-weaned Simmental calves

As shown in Table 5, at 120 d, the GH level of SFC33 group was extremely significantly higher than the other three groups (*P* < 0.01), and the difference between the three groups was not significant (*P* > 0.05). At 40 d, the INS levels of the three experimental groups were significantly higher than those of the CK group (*P* < 0.05); at 120 d, the SFC33 and SFC100 groups were extremely significantly higher than those of the CK and SFC66 groups (*P* < 0.01). At 80 d, the IGF-1 level was significantly higher in the SFC100 group than in the CK group (*P* < 0.05); at 120 d, it was extremely significantly higher in the SFC33 group than in the SFC66 and SFC100 groups (*P* < 0.01), and extremely significantly higher in the CK group than in the SFC100 group (*P* < 0.01). At 40 d, IGF-2 levels were highly significantly higher in the CK and SFC100 groups than in the SFC66 group (*P* < 0.01); at 80 d, they were highly significantly higher in the SFC66 group than in the SFC33 group (*P* < 0.01), and significantly higher in the CK group than in the SFC33 group (*P* < 0.05); and at 120 d, they were highly significantly higher in the SFC66 group than in the CK group and the SFC33 group (*P* < 0.01). The results of the experiment showed that feeding 33% steam-flaked corn-based starter to pre-weaned calves significantly increased their serum levels of growth hormone (GH), insulin-like growth factor-1 (IGF-1), insulin-like growth factor-2 (IGF-2), and insulin (INS). There was a highly significant interaction effect between group and time for GH, INS, IGF-1 and IGF-2 (*P* < 0.01).

**Table 5:** Effect of feeding different proportions of steam-flaked corn-based starter on hormonal changes in pre-weaned Simmental calves.

**Item^a^**	**Days**	**Group**				***P*** **-value**		
		**CK**	**SFC33**	**SFC66**	**SFC100**	**Group**	**Days**	**Group** × **time**
GH (ng/ml)	0 d	3.49 ± 0.16	4.34 ± 0.51	4.11 ± 0.45	3.22 ± 0.56	0.053	0.000	0.002
	40 d	4.62 ± 0.36	4.78 ± 0.63	4.64 ± 0.54	4.49 ± 075	0.808		
	80 d	6.67 ± 1.75	6.06 ± 1.66	6.02 ± 0.81	6.35 ± 0.66	0.733		
	120 d	6.49 ± 1.71^B^	8.87 ± 0.40^A^	6.38 ± 1.17^B^	6.41 ± 1.76^B^	0.002		
INS (uIU/ml)	0 d	14.17 ± 0.78	13.30 ± 0.61	13.42 ± 1.17	13.86 ± 0.58	0.815	0.000	0.003
	40 d	13.18 ± 1.70^b^	14.39 ± 0.73^a^	14.54 ± 0.44^a^	14.49 ± 1.37^a^	0.083		
	80 d	15.37 ± 0.38	15.37 ± 1.46	15.04 ± 0.71	15.10 ± 0.62	0.815		
	120 d	14.11 ± 0.26^B^	15.50 ± 0.92^A^	14.12 ± 0.39^B^	15.13 ± 0.58^A^	0.000		
IGF-1 (ng/ml)	0 d	201.61 ± 12.80	203.95 ± 7.08	191.74 ± 15.45	186.95 ± 20.27	0.088	0.000	0.000
	40 d	216.92 ± 4.71	219.31 ± 26.16	219.92 ± 21.97	226.34 ± 35.27	0.886		
	80 d	236.21 ± 9.41^b^	247.50 ± 10.00^ab^	250.12 ± 15.66^ab^	255.13 ± 21.90^a^	0.105		
	120 d	288.66 ± 13.13^ABb^	313.29 ± 23.99^Aa^	270.75 ± 22.11^BCbc^	259.54 ± 13.16^Cc^	0.000		
IGF-2 (ng/ml)	0 d	415.13 ± 34.76	431.42 ± 27.55	423.10 ± 20.82	422.08 ± 67.30	0.072	0.000	0.000
	40 d	435.08 ± 34.46^Aa^	395.16 ± 29.30^ABb^	368.32 ± 33.90^Bb^	434.93 ± 27.38^Aa^	0.000		
	80 d	357.66 ± 4.31^ABa^	336.23 ± 12.33^Bb^	369.40 ± 17.95^Aa^	353.60 ± 28.40^ABab^	0.009		
	120 d	313.10 ± 13.10^B^	312.39 ± 31.10^B^	362.53 ± 44.64^A^	336.51 ± 7.14^AB^	0.004		

^a^CK, control check, 0% steam-flaked corn was used to replace the original crushed corn in the starter. SFC33: 33% steam-flaked corn was used to replace the original crushed corn in the starter. SFC66: 66% steam-flaked corn was used to replace the original crushed corn in the starter diet. SFC100: 100% steam-flaked corn was used to replace the original crushed corn in the starter diet. the results were expressed as mean ± standard deviation, *n* = 8.

^A, B, C^Indicates that the difference between indicators in the same row is highly significant (*P* < 0.01).

^a, b, c^Indicates that the difference between indicators in the same row is significant (*P* < 0.05). Only comparisons between the same rows are performed.

GH, growth hormone; INS, insulin; IGF-1, insulin-like growth factor 1; IGF-2, insulin-like growth factor 2.

### 3.5 Effect of feeding different proportions of steam-flaked corn-based starter on serum nutrient metabolism in pre-weaned Simmental calves

#### 3.5.1 Multidimensional parsing and visualization of metabolomics data

**Figure 2:**
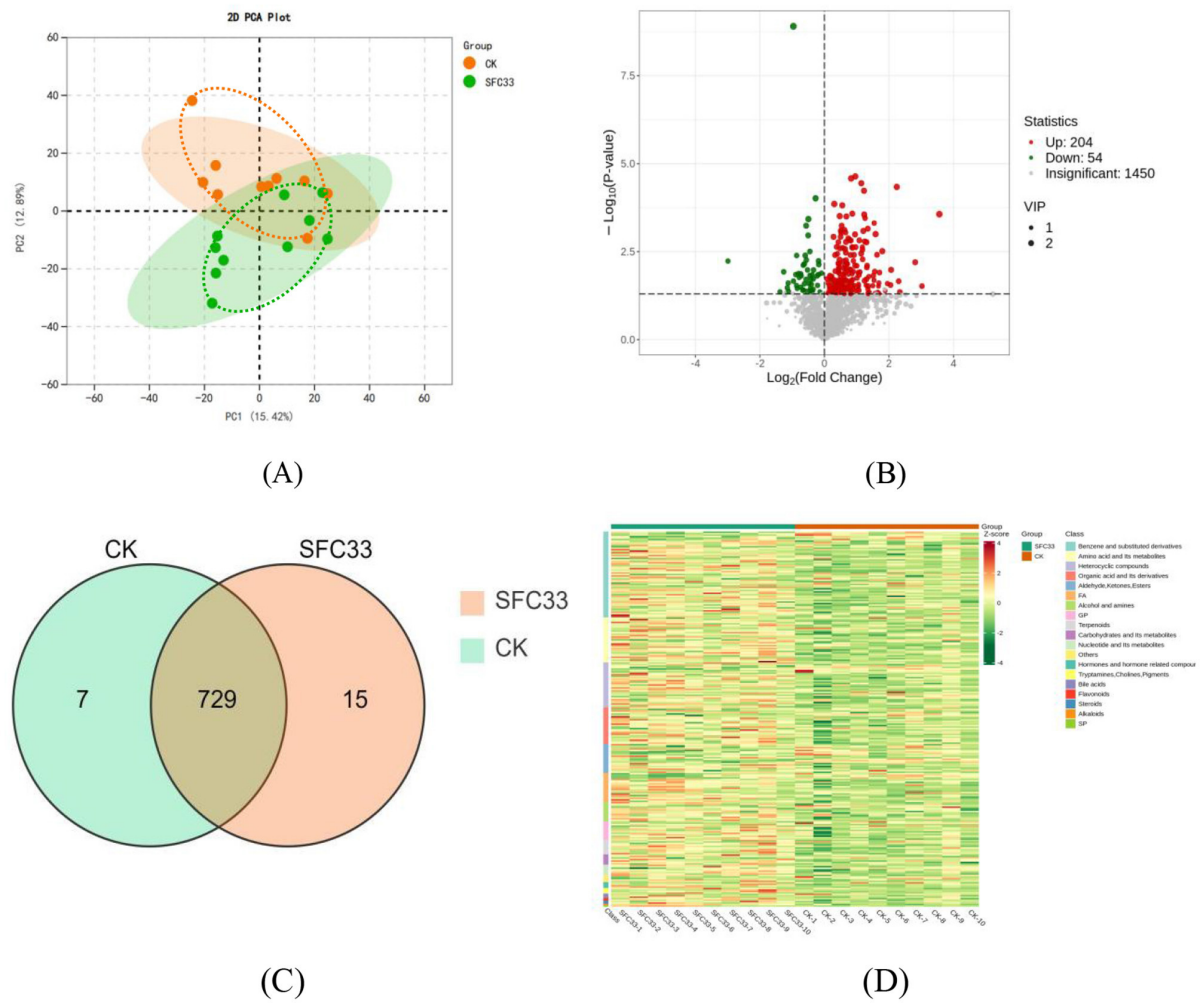
Differential metabolite analysis chart. **(A)** Plot of principal component analysis of the total sample. PC1 denotes the first principal component, PC2 denotes the second principal component, and the percentage denotes the rate of interpretation of the data set by that principal component; each point in the graph denotes a sample, and samples in the same group are denoted by using the same color, and Group is the subgroup. **(B)** SFC33 VS CK differential metabolite volcano map. Each dot in the volcano diagram represents a metabolite, in which green dots represent down-regulated differential metabolites, red dots represent up-regulated differential metabolites, and gray dots represent metabolites detected but with insignificant differences: the horizontal coordinate represents the logarithm of the multiplicity of the difference of the relative content of a metabolite between the two groups of samples (log_2_FC), and the bigger the absolute value of the horizontal coordinate is, the bigger is the difference of relative content of the substance between the two groups of samples. Under the VIP + FC + *P*-value screening condition: the vertical coordinate indicates the significance level of difference (–log_10_*P*-value), and the size of the dot represents the VIP value. **(C)** SFC33 VS CK differential metabolite Wayne diagram. Each circle in the figure represents a comparison group, and the number of overlapping circles represents the number of differential metabolites common to the comparison groups, while the number without overlapping circles represents the number of differential metabolites specific to the comparison groups. **(D)** SFC33 VS CK differential metabolite clustering heat map. Horizontal is the sample information, vertical is the differential metabolite information, group is the grouping, and different colors are filled with different values obtained after normalization for different relative contents (red for high content, green for low content). The clustered lines on the left side of the figure are metabolite clusters, and the clustered lines at the top of the figure are sample clusters.

As shown in the Figure 2, the first principal coordinate of serum metabolic samples (PC1), distinguished the serum metabolic differentials with a contribution of 12.4%, and the second principal coordinate (PC2) with a contribution of 10.94%. In this experiment, the samples of each group were more discrete with lower similarity, which indicated that the feeding of steam-flaked corn-based starter had a significant effect on the metabolic differentials in serum of the pre-weaned calves (*P* < 0.05) (Figure 2A). The data were first standardized by unit variance scaling (UV), and all the samples were analyzed by clustering heatmap, and the clustering heatmap was plotted by using the R program script, in which there were 258 metabolically variant substances, of which 204 were up-regulated and 54 were down-regulated (Figure 2B). As shown in the Wayne diagram, a total of 751 metabolites were detected in the SFC33 VS CK group, of which 729 metabolites were common to both groups, seven metabolites were specific to the CK group, and 15 metabolites were specific to the SFC33 group (Figure 2C). The two groups of differential metabolites clustered well as shown by the differential metabolite heat map (Figure 2D).

#### 3.5.2 Results of KEGG enrichment analysis of differential metabolites

**Figure 3:**
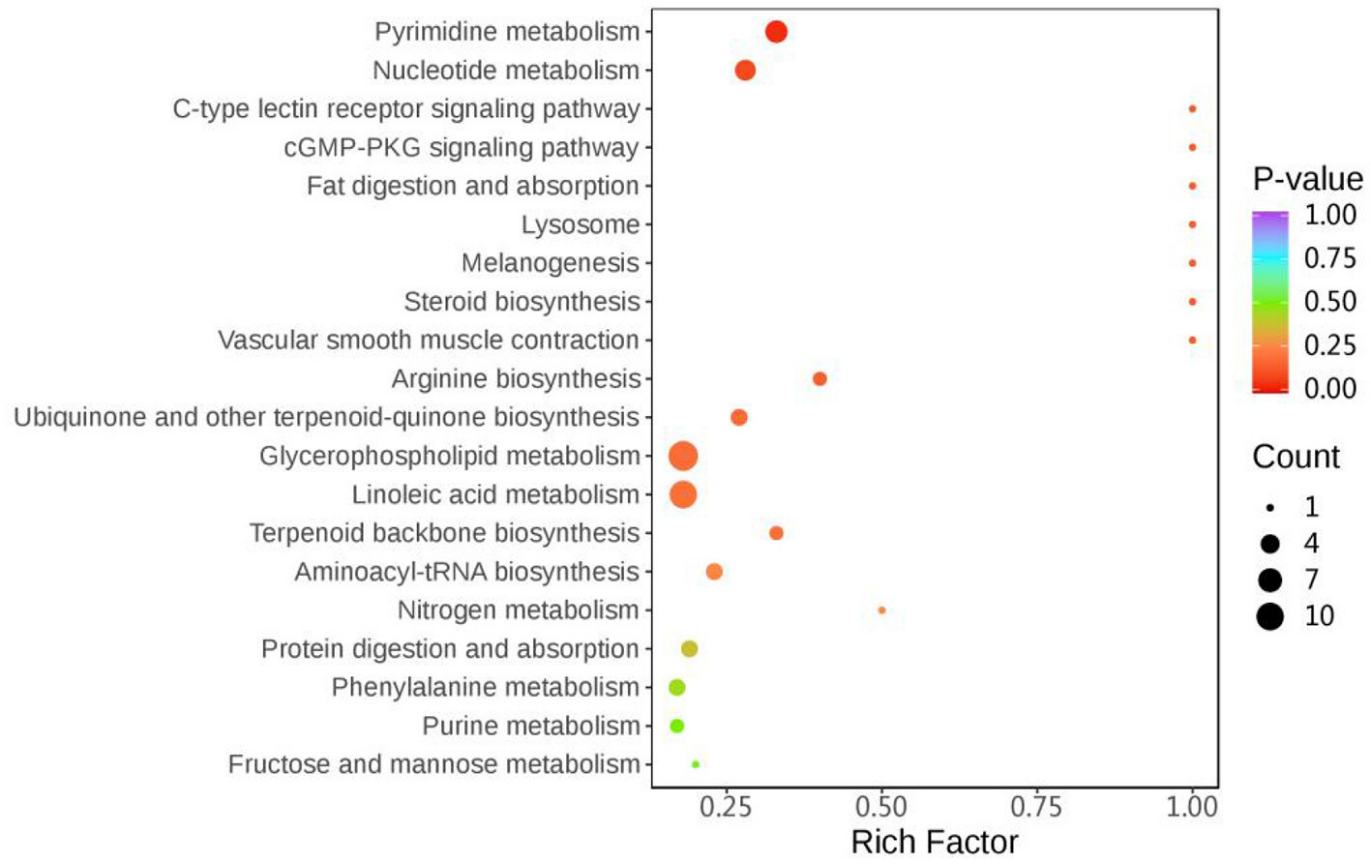
SFC33 VS CK metabolic pathway bubble map. The horizontal coordinate indicates the Rich Factor corresponding to each pathway, the vertical coordinate is the pathway name (sorted by *P*-value), and the color of the dots is the size of the *P*-value, the redder indicates the more significant enrichment. The color of the dot is the size of the *P*-value, the redder the more significant the enrichment. The size of the dot represents the number of different metabolites enriched.

The results of KEGG enrichment analysis of differential metabolites for the first 20 pathways are shown in Figure 3, respectively: Phenylalanine metabolism, Nucleotide metabolism, Vascular smooth muscle contraction, Steroid biosynthesis, Melanogenesis, Lysosome, Fat digestion and absorption, cGMP-PKG signaling pathway, C-type lectin receptor signaling pathway, Arginine biosynthesis, Ubiquinone and other terpenoid-quinone biosynthesis, Glycerophospholipid metabolism, Linoleic acid metabolism, Terpenoid backbone biosynthesis, Pyrimidine metabolism, Aminoacyl-tRNA biosynthesis, Nitrogen metabolism, Protein digestion and absorption, Purine metabolism, Fructose and mannose metabolism.

#### 3.5.3 Differential metabolite screening results

As shown in Table 6, a total of 31 differential metabolites were screened in combination with differential metabolic pathways, of which 27 were up-regulated and four were down-regulated.

**Table 6:** SFC33 VS CK differential metabolic pathways and differential metabolites.

**Metabolic pathway**	***P*-value**	**Differential metabolites**	**VIP**	***P*-value**
Pyrimidine metabolism	0.0231	*L-Glutamine*↑	1.3301	0.0218
		*L-Hydrogenated orotate*↑	2.1694	0.0012
		*Uracil nucleoside*↑	2.0480	0.0008
		*Uracil*↑	1.4406	0.0260
		*Cytosine*↑	1.5935	0.0109
		*Pseudouridine*↑	2.5250	0.0001
Nucleotide metabolism	0.0792	*L-Glutamine*↑	1.3301	0.0218
		*Uracil nucleoside*↑	2.0480	0.0008
		*Uracil*↑	1.4406	0.0260
		*Cytosine*↑	1.5935	0.0109
Vascular smooth muscle contraction	0.1347	*Adenosine*↓	1.0882	0.0401
Steroid biosynthesis	0.1347	*Triglyceride*↑	2.2483	0.0013
Melanogenesis	0.1347	*L-Tyrosine*↑	2.1068	0.0012
lysosome	0.1347	*Mannose*↑	1.3320	0.0424
Fat digestion and absorption	0.1347	*Triglyceride*↑	2.2483	0.0013
cGMP-PKG signaling pathway	0.1347	*Adenosine*↓	1.0882	0.0401
C-type lectin receptor signaling pathway	0.1347	*Mannose*↑	1.3320	0.0424
Biosynthesis of arginine	0.1362	*L-Glutamine*↑	1.3301	0.0218
		*L-Citrulline*↑	1.6664	0.0321
Biosynthesis of ubiquinone and other terpene-quinones	0.1738	*L-Tyrosine*↑	2.1068	0.0012
		*Phytodiphosphoric acid*↑	1.3079	0.0314
		*6-Geranylgeranyl-2,3-dimethylbenzenediol*↑	1.6162	0.0364
Glycerophospholipid metabolism	0.1789	*1,2-Dioleoyl-sn-glycero-3-phosphatidylcholine*↓	1.5601	0.0154
		LPC(18:1/0:0)↓	1.6420	0.0324
		PC(6:0/6:0)↑	2.7023	0.0003
		LPC(14:0/0:0)↑	1.3045	0.0258
		LPC(16:2/0:0)↑	1.1722	0.0319
		*1-Myristoyl-2-stearoyl-sn-glycero-3-phosphocholine*↑	1.5107	0.0207
		LPE(16:0/0:0)↑	1.5879	0.0186
		LPC(0:0/18:2)↑	1.3192	0.0363
		*Glycerophosphoric acid-N-arachidonic acid ethanolamine*↑	2.0064	0.0015
		LPE(0:0/18:3)↑	1.2534	0.0436
		LPE(0:0/18:2)↑	1.7946	0.0288
		PC(18:1(11Z)/14:1(9Z))↑	1.7955	0.0004
Linoleic acid metabolism	0.1843	LPC(16:2/0:0)↑	1.1722	0.0319
		*1,2-Dioleoyl-sn-glycero-3-phosphatidylcholine*↑	1.5601	0.0154
		LPC(18:1/0:0)↓	1.6420	0.0324
		PC(6:0/6:0)↑	2.7023	0.0003
		LPC(14:0/0:0)↑	1.3045	0.0258
		*9,12,13-Trihydroxy-octadecadienoic acid*↑	2.0448	0.0056
		*1-Myristoyl-2-stearoyl-sn-glycero-3-phosphocholine*↑	1.5107	0.0207
		*Gamma-linolenic acid*↑	1.6605	0.0094
		*13-Peroxyhydroxy-9Z,11E-octadecadienoic acid*↑	1.8356	0.0133
		PC(18:1(11Z)/14:1(9Z))↑	1.7955	0.0004
Biosynthesis of terpene skeletons	0.1872	*3,5-Dihydroxy-3-methylpentanoic acid*↑	2.0345	0.0024
		*Phytodiphosphoric acid*↑	1.3079	0.0314
Biosynthesis of aminoacyl-tRNA	0.2489	*L-Glutamine*↑	1.3301	0.0218
		*L-Methionine*↑	2.1068	0.0012
		*L-Tyrosine*↑	2.1068	0.0012
Nitrogen metabolism	0.2515	*L-Glutamine*↑	1.3301	0.0218
Protein digestion and absorption	0.3679	*L-Glutamine*↑	1.3301	0.0218
		*L-Tyrosine*↑	2.1068	0.0012
		Methionine↑	1.8357	0.0214
Phenylalanine metabolism	0.4463	*DL-3-Phenyl lactic acid*↓	1.3510	0.0142
		*2-Hydroxyphenylacetic acid*↑	2.0556	0.0079
		*L-Tyrosine*↑	2.1068	0.0012
Purine metabolism	0.4971	*L-Glutamine*↑	1.3301	0.0218
		*Adenosine*↓	1.0882	0.0401
Fructose and mannose metabolism	0.5167	*Mannose*↑	1.3320	0.0424

↑ Indicates that the differential metabolite is up-regulated; ↓ indicates that the differential metabolite is down-regulated, the results were not statistically significant (0.05 < *P*), the results were statistically significant (0.01 < *P* < 0.05), the results were highly statistically significant (*P* < 0.01), VIP: OPLS-DA was calculated to be proportional to the contribution of metabolites. LPC, lysophosphatidylcholine; LPE, lysophosphatidylethanolamine.

## 4 Discussion

### 4.1 Effect of feeding different proportions of steam-flaked corn-based starter on daily weight gain of pre-weaned Simmental calves

The results of this experiment showed that fedding 33% steam-flaked corn-based starter could enhance the growth performance of pre-weaned calves. However, as the proportion of steam-flaked corn in the starter increased, calves' daily weight gain decreased and was lower than the control group fed regular crushed corn. In addition, Zhang et al. also found that the daily weight gain of calves did not change significantly when steam-flaked corn completely replaced regular crushed corn in the starter in a trial with Holstein male calves, which is consistent with our experimental results (10).

Although higher percentages of steam-flaked corn replacement have been commonly found to promote increases in daily weight gain in studies on adult beef cattle (11, 12), this effect may not be applicable in pre-weaned calves. The reason for this is that pre-weaned calves differ significantly from adults in their digestive patterns. Specifically, the rumen of calves is not yet fully developed and their main source of nutrition is breast milk. In addition, the calf's esophageal groove reflex mechanism directs sucked breast milk directly to the abomasum for digestion, while the remainder enters the small intestine for further digestion and absorption (13). These physiological characteristics may have influenced the efficiency of utilization of steam-flaked corn in pre-weaned calves, leading to a growth performance different from the results of previous studies.

### 4.2 Effect of feeding different proportions of steam-flaked corn-based starter on serum biochemical indices in pre-weaned Simmental calves

Total protein (TP) in serum is mainly composed of two major components, albumin (ALB) and globulin (GLB). The total protein level is an important indicator of the immune status of the body, which is usually maintained in a dynamic equilibrium and is not prone to significant fluctuations (14). When globulin synthesis moderately increases or albumin synthesis moderately decreases, it may signal a trend toward improved body immune function (15). Total protein and blood urea nitrogen (BUN) levels together reflect the body's digestion and utilization of protein. Specifically, the total protein level is positively correlated with the intake of protein feed, while the blood urea nitrogen level is inversely correlated with the body's ability to digest and absorb protein (16). Compared with previous studies, feeding 33% steam- flaked corn starter effectively increased the total protein content and reduced the BUN content to some extent, indicating that feeding 33% steam- flaked corn starter can effectively improve the protein absorption capacity of calves (17). Triglycerides (TG) are fat molecules formed by combining long-chain fatty acids with glycerol, while total cholesterol (TC) represents the total amount of all lipoproteins in the blood. These two substances play a key role in the storage and utilization of fat in the body, and their content levels directly reflect the body's ability to absorb and metabolize lipids (18, 19). Lipid metabolism is particularly important for calves during the growth and development period in order to meet the large amount of energy and nutrients required for rapid growth. In the present experiment, serum total cholesterol level increased although the serum triglyceride content of calves did not change significantly. This result suggests that feeding 33% steam-flaked corn-based starter for pre-weaned calves was able to utilize lipid resources more efficiently, especially cholesterol, thus providing more energy support during the rapid growth phase before weaning, which in turn may have contributed to the pre-weaned calves' daily weight gain.

Glucose (GLU) in the blood is the direct substance for energy conversion in the organism and the source of energy for growth, development, and all metabolic activities. The level of its concentration has a crucial influence on maintaining the normal function and growth of the organism. When the blood glucose level decreases, it directly affects the immune defense of the organism (20). In the present experiment, we observed a significant increase in the blood glucose level of calves in the test group as compared to the control group and also as compared to the study of Wan et al. (21). This finding suggests that the feeding steam-flaked corn-based starter can effectively promote the absorption and utilization of glucose in pre-weaned calves, thus providing more adequate energy support for calf growth.

### 4.3 Effect of feeding different proportions of steam-flaked corn-based starter on immune factors in pre-weaned Simmental calves

Immunoglobulins mainly include three types of Ig-A, Ig-M, and Ig-G, which are the core indicators for assessing the strength of immune function in calves (22). In this experiment, we noticed that all three immunoglobulin indices of the SFC33 group of calves were improved, and all of them were also improved to some extent compared with the previous study (23), indicating enhanced immune ability. However, for the calves in the other two experimental groups, the immunoglobulin levels decreased to different degrees. In response to this phenomenon, we hypothesized that it might be due to the high addition of steam-flaked corn in these two experimental groups's starter, which in turn led to a significant increase in the proportion of concentrate feed in the diet composition. This change may have placed an additional burden on the digestive and metabolic systems of the calves, affecting their adequate absorption and utilization of nutrients. Ultimately, this decrease in nutrient absorption and utilization may have led to a weakening of immune function and showing a trend of reduced immunity in pre-weaned calves.

### 4.4 Effect of feeding different proportions of steam-flaked corn-based starter on hormonal changes in pre-weaned Simmental calves

Growth hormone (GH), secreted mainly by the pituitary gland, plays multiple critical roles in the organism. It not only promotes protein synthesis, accelerates lipid metabolism and glucose metabolism processes, but also functions to stimulate the secretion of insulin-like growth factor 1 (IGF-1), IGF-1 is a kind of alkaline single-chained polypeptide produced locally in the liver and other tissues of animals, is a key physiological regulator of growth hormone (GH) *invivo*, and is therefore widely known as an intermediary of growth (25–27). IGF-1 exerts its function in the form of autocrine or paracrine by binding to related proteins in the interstitial fluid of tissues, thus finely regulating the normal growth and energy metabolism process of the organism. In this experiment, the serum levels of growth hormone (GH) and insulin-like growth factor 1 (IGF-1) were significantly higher in calves of the SFC33 group than in the other groups, and there was a more effective increase in the level of GH, although there was little change in the level of IGF-1 compared with that of the previous group (24). This finding suggests that feeding 33% steam-flaked corn-based starter has a significant promotion effect on pre-weaning calves, which not only accelerates their growth and development but also enhances the body's ability to metabolize nutrients, thus indirectly enhancing the growth performance of pre-weaned calves.

Insulin (INS) is the only hormone in the organism responsible for lowering blood glucose levels, and it also promotes the process of glycogen, lipids and protein synthesis (28). In this experiment, when the experiment was carried out to the 120th day, we found that the insulin levels of all the calves in the experimental group were significantly higher than those of the control group, and there was also a certain degree of increase in comparison with the previous study (24). This result suggests that feeding steam-flaked corn-based starter helps to stimulate insulin secretion, which in turn helps to regulate glucose homeostasis in the organism in pre-weaned calves.

Insulin-like growth factor 2 (IGF-2) plays an important role in organisms by increasing amino acid utilization in protein synthesis, inhibiting protein degradation, and promoting the proliferation of bone and muscle cells (29). In this experiment, we observed a corresponding increase in IGF-2 levels with the addition of steam-pressed maize, and a more pronounced increase in IGF-2 levels compared to previous studies (29). This finding suggests that feeding steam-flaked corn-based can effectively enhance the utilization of amino acids, and promote the growth of bone and muscle tissues by pre-weaned calves.

### 4.5 Enrichment of KEGG, a serum differential metabolite, in pre-weaned Simmental calves by different proportions of steam-flaked corn-based starter

Phenylalanine, as one of the indispensable amino acids in the organism, is a key element in the process of protein metabolism, and its main area of utilization is concentrated around the hepatic portal vein in animals, which has a crucial impact on the growth and development of animals. Calves can effectively utilize phenylalanine to promote the process of protein synthesis and metabolism, thus guaranteeing the healthy development of muscles and bones in the body (30). In this experiment, by analyzing the KEGG pathway enrichment between the SFC33 group and the CK group, We observed a *P*-value of 0.4463 for the phenylalanine metabolic pathway, a result that suggests that feeding 33% steam-flaked corn starter could somewhat increase phenylalanine concentrations and thus improve protein digestion and absorption efficiency in pre-weaned calves.

Fructose and mannose, as common monosaccharide components in animals, are not only involved in the process of glucose metabolism but also has been found to help promote insulin secretion (31). In this experiment, the KEGG pathway enrichment results of the SFC33 and CK groups showed a *P*-value of 0.5167 for the fructose and mannose metabolic pathways, suggesting that feeding 33% steam-flaked corn starter could somewhat improve the absorption and utilization of these two sugars in pre-weaned calves.

Glycerophospholipids, as the main building blocks of cell membranes in calves, are not only crucial for maintaining the stability of cell membranes and the balance of the internal environment, but also a key link in lipid metabolism, which provides the necessary energy support for calf growth and development, and plays a positive role in enhancing the immune function of the body (32, 33). In this experiment, the KEGG pathway enrichment analysis of the SFC33 and CK groups showed that the *P*-value of the glycerophospholipid metabolic pathway was 0.1789, which implies that feeding 33% of steam-flaked corn starter can increase the content of glycerophospholipids to some extent in pre-weaned calves, thereby promoting their metabolic uptake of lipids.

### 4.6 Effect of different proportions of steam-flaked corn-based starter starter on serum nutrient metabolism in pre-weaned Simmental calves

Although the important pathways mentioned above were not statistically significantly enriched, there was a significant increase in all of the important differential metabolites in them, and we speculate that it may be due to the fact that these key pathways are relatively large, that the elevation of these few differential metabolites was not able to bring about a statistically significant increase in the metabolic pathways in which they are involved, but they still had some degree of effect on the pathways.

#### 4.6.1 Metabolism of protein and amino acid substances

Glutamine, as a core element of energy production, protein, and nucleic acid synthesis, has a crucial supporting role in metabolic homeostasis, structural integrity, and functional performance of the small intestine. It is not only the basic substance for energy metabolism in intestinal mucosal cells but also an indispensable raw material for protein synthesis (34). For pre-weaned calves, breast milk is an important source of glutamine, and once weaned, the amount of glutamine synthesized in the calf's body often fails to meet the demand, which in turn affects the intestinal function and reduces the efficiency of feed digestion (35). The results of this experiment showed that compared with the CK group, the L-glutamine level in the serum of calves in the SFC33 group increased significantly and was enriched in multiple pathways such as nucleotide metabolism, arginine biosynthesis, nitrogen metabolism, purine metabolism, and protein digestion and absorption, which strongly proved that feeding 33% steam-flaked corn-based starter could enhance the digestion and utilization of proteins by pre-weaned calves, and effectively alleviate the weaning-borne stress response.

Tyrosine, as a key component of protein synthesis, plays a central role in driving the development of muscle, bone, and other tissues in calves. An adequate supply of tyrosine helps calves synthesize high-quality proteins to meet their rapid growth requirements (36, 37). In addition, tyrosine is involved in the process of melanin synthesis, which has a direct impact on the calf's coat color and skin health. As an important pigment in animals, the normal synthesis of melanin depends on an adequate supply of tyrosine (38). In this experiment, it was found that compared with the CK group, the serum L-tyrosine content of calves in the SFC33 group was significantly increased and enriched in key pathways such as phenylalanine metabolism, aminoacyl-tRNA biosynthesis, and protein digestion and absorption, which suggests that feeding steam-flaked corn-based starter can help to promote the digestion and absorption of proteins in pre-weaned calves, as well as maintain their skin health and normal coat color.

#### 4.6.2 Sugar metabolism

Mannose has a significant effect in elevating the serum glucose level of calves, which not only provides a rich resource of sugar for calves but also plays a role in the immune system by participating in the synthesis of glycoproteins, which in turn enhances the immunity of calves (38). The results of the present experiment showed that calves in the SFC33 group fed 33% steam-flaked corn-based starter had significantly higher serum levels of mannose and enrichment was observed in the metabolic pathway of fructose and mannose as compared to the control group (CK group). This finding suggests that feeding 33% steam-flaked corn-based starter helps to promote the digestion and absorption of sugars in pre-weaned calves.

#### 4.6.3 Lipid metabolism

A diverse group of Lysophosphatidylcholine (LPC) molecules exists in the organism, with major differences in the length of the carbon chain and the number of double bonds. These LPC molecules play a key role in the organism and are mainly involved in lipid metabolism processes. Wang et al. demonstrated that there was a significant difference in the levels of LPC in serum samples from obese and normal populations, further confirming the negative correlation between LPC levels and body weight (39, 40). In the present experiment, changes in several phospholipid acyl molecules, including up- and down-regulation phenomena, were detected in the serum of calves from the SFC33 group compared to the CK group. These changes were particularly significant in the glycerophospholipid metabolism pathway as well as the linoleic acid metabolism pathway, suggesting that lipid metabolism in the organism is in a state of dynamic balance adjustment.

#### 4.6.4 Metabolism of other substances

Cytosine, as a core component in the structure of DNA, is closely paired with guanine, and together they maintain the stability of the double helix structure of DNA, which is crucial for the accuracy and durability of genetic information. During calf growth and development, an adequate supply of cytosine plays a key role in ensuring that cells can divide and proliferate normally, thereby promoting the overall growth and development of the calf (41). Similarly, uracil, as a key component of RNA, pairs with adenine and is an indispensable part of RNA synthesis, which is responsible for transmitting genetic information and directing protein synthesis in calves (42), so an adequate supply of uracil is important for normal protein synthesis and metabolism in calves, which can help to maintain the normal physiological functions of calves and promote their growth and development. The results of the present experiment showed that the uracil content in the calves was higher than that in the calves. The results of this experiment showed that both cytosine and uracil contents in the serum of calves in the SFC33 group were significantly up-regulated compared with those in the CK group, and these changes were significantly enriched in nucleotide metabolism and pyrimidine metabolism pathways. This finding suggests that feeding 33% steam-flaked corn-based starter may contribute to pre-weaned calf growth and development and protein synthesis processes by promoting the activity of these key metabolic pathways.

## 5 Conclusion

Feeding 33% steam-flaked corn starter to pre-weaned Simmental calves significantly improved calf growth performance, enhanced immune function, and effectively promoted the metabolism of nutrients such as sugars, lipids, and proteins. In this process, the pyrimidine metabolic pathway was significantly enriched, and the contents of metabolically differentiated substances L-glutamine, L-tyrosine, mannose, and LPC (16:2/0:0) were significantly increased. In conclusion, the present study provides a theoretical basis for breeding healthy and high-quality calves, as well as basic support for the study of nutritional metabolism in young animals in animal husbandry.

## Data Availability

The datasets presented in this study can be found in online repositories. The names of the repository/repositories and accession number(s) can be found below: https://db.cngb.org/search/project/CNP0006234/, China National GeneBank DataBase, CNGBdb CNP0006234.
